# Association between triglyceride-glucose index and the risk of cardiometabolic diseases in metabolically healthy obese individuals: a prospective cohort study

**DOI:** 10.3389/fendo.2025.1524786

**Published:** 2025-05-19

**Authors:** Yanjuan Chen, Weiqiang Wu, Zefeng Cai, Kuangyi Wu, Huancong Zheng, Peng Fu, Yuxian Wang, Xianxuan Wang, Yulong Lan, Shuohua Chen, Shouling Wu, Youren Chen

**Affiliations:** ^1^ Department of Endocrinology, Second Affiliated Hospital of Shantou University Medical College, Shantou, China; ^2^ Department of Cardiology, Second Affiliated Hospital of Shantou University Medical College, Shantou, China; ^3^ Department of Physiology, Temerty Faculty of Medicine, University of Toronto, Toronto, ON, Canada; ^4^ Department of Cardiology, Kailuan General Hospital, Tangshan, China

**Keywords:** triglyceride-glucose index, metabolically healthy obese, cardiometabolic disease, diabetes, stroke, coronary heart disease

## Abstract

**Background:**

Metabolically healthy obese (MHO) individuals meet the criteria for obesity with normal blood glucose and lipid metabolism parameters, absence of hypertension, and no concurrent cardiovascular diseases. However, the association between the triglyceride-glucose (TyG) index and the risk of cardiometabolic disease (CMD) in MHO individuals remains unclear.

**Methods and results:**

This study included obese individuals who underwent health examinations at Kailuan Group from 2006 to 2010, whom without a history of hypertension, diabetes, hyperlipidemia, cardiovascular disease, as the study participants. A total of 4750 participants were included in this study. The TyG index was calculated as ln[TG (mg/dL) × FPG (mg/dL)/2] and divided into four groups based on quartiles: Q1 group (<8.18); Q2 group (8.18-8.41); Q3 group (8.42-8.62); Q4 group (≥8.63). The Cox proportional hazards model was used to assess the relationship between the TyG index and risk of CMD incidence. During a median follow-up period of 11 (IQR 10.3, 11.2) years, 826 participants experienced CMD, among whom 131 participants developed coronary heart disease, 215 participants developed stroke, and 542 participants developed diabetes. After adjusting for multiple confounding factors, compared with the Q1 group, the adjusted HRs (95% CI) for CMD in the Q2-Q4 groups were 1.33 (1.03, 1.65), 1.37 (1.04, 1.82), and 2.04 (1.56, 2.68) (*P*<0.0001). A similar trend was found in the subtypes of CMD in coronary heart disease, stroke, and diabetes. Restrictive cubic spline analysis revealed a linear dose-response relationship between the TyG index and the risk of CMD.

**Conclusions:**

A high TyG index increases the risk of CMD in MHO individuals. Monitoring and maintaining an appropriate TyG index may contribute to the prevention of CMD risk in MHO individuals.

## 1 Introduction

Over the past four decades, the global prevalence of obesity has nearly doubled, reaching pandemic proportions, with over 878 million adults being affected by obesity as of 2022 (1). The widespread issue of obesity imposes a significant burden on both individual health and socioeconomic systems (2). Cardiometabolic diseases (CMD), including type 2 diabetes, coronary heart disease, and stroke (3, 4) have been on the rise. Studies indicate that individuals aged 60 years and above with CMD experience a reduced life expectancy of 6–10 years compared to those without CMD, and those with two or more CMDs experience a life expectancy reduction of over 15 years (5). Obesity exacerbates metabolic risk factors (hypertension, hyperglycemia, dyslipidemia) and directly contributes to cardiac and cerebral dysfunction (3, 6–8). Notably, obesity-driven metabolic dysfunction-associated steatotic liver disease (MASLD) promotes systemic insulin resistance (IR) and inflammation via hepatic lipid accumulation (9), further amplifying CMD risk (10, 11). These intertwined mechanisms underscore the urgency of addressing obesity-related CMD (3, 8, 10–12).

However, not all obese individuals experience metabolic disruptions or develop cardiovascular diseases (13, 14). A subset of obese individuals is referred to as metabolically healthy obese (MHO) (14–16). MHO is a concept based on clinical observations, and although it lacks a precise definition, normal blood glucose and lipid metabolism parameters, absence of hypertension, and no concurrent cardiovascular diseases are often considered criteria for diagnosing MHO (17–19). However, because individuals in the MHO group do not manifest metabolic abnormalities, identifying high-risk individuals prone to CMD within the MHO population has become a noteworthy issue. Some studies suggest that, compared to metabolically healthy non-obese (MHNO) individuals, MHO individuals primarily exhibit ectopic fat deposition (visceral and hepatic) and biological mechanisms such as IR (15). The triglyceride-glucose (TyG) index, a validated surrogate marker of insulin resistance (20, 21), integrates lipid and glucose metabolism dysregulation. Elevated TyG correlates with mitochondrial dysfunction, oxidative stress, and systemic inflammation, driving endothelial injury and atherosclerosis (20, 22–24). In obesity, ectopic hepatic fat exacerbates IR via dysregulated gluconeogenesis and VLDL overproduction, establishing a self-reinforcing cycle of TyG elevation and metabolic deterioration (15, 16, 25). Thus, identifying TyG-driven metabolic vulnerability in MHO populations may mitigate CMD progression.

Despite the absence of traditional risk factors, MHO individuals exhibit ectopic fat deposition and subtle insulin resistance (15, 25), creating a latent vulnerability to cardiometabolic disease. The TyG index, a proxy for adipose-liver crosstalk (20, 22), may detect this vulnerability earlier than conventional biomarkers. No prior study has evaluated TyG’s prognostic value in rigorously defined MHO populations, representing a critical gap our work addresses (26, 27). Leveraging the longitudinal Kailuan study, this investigation aims to clarify the association between the TyG index and CMD risk in MHO individuals, offering insights for early risk stratification.

## 2 Methods

### 2.1 Study design and participants

**Figure 1:**
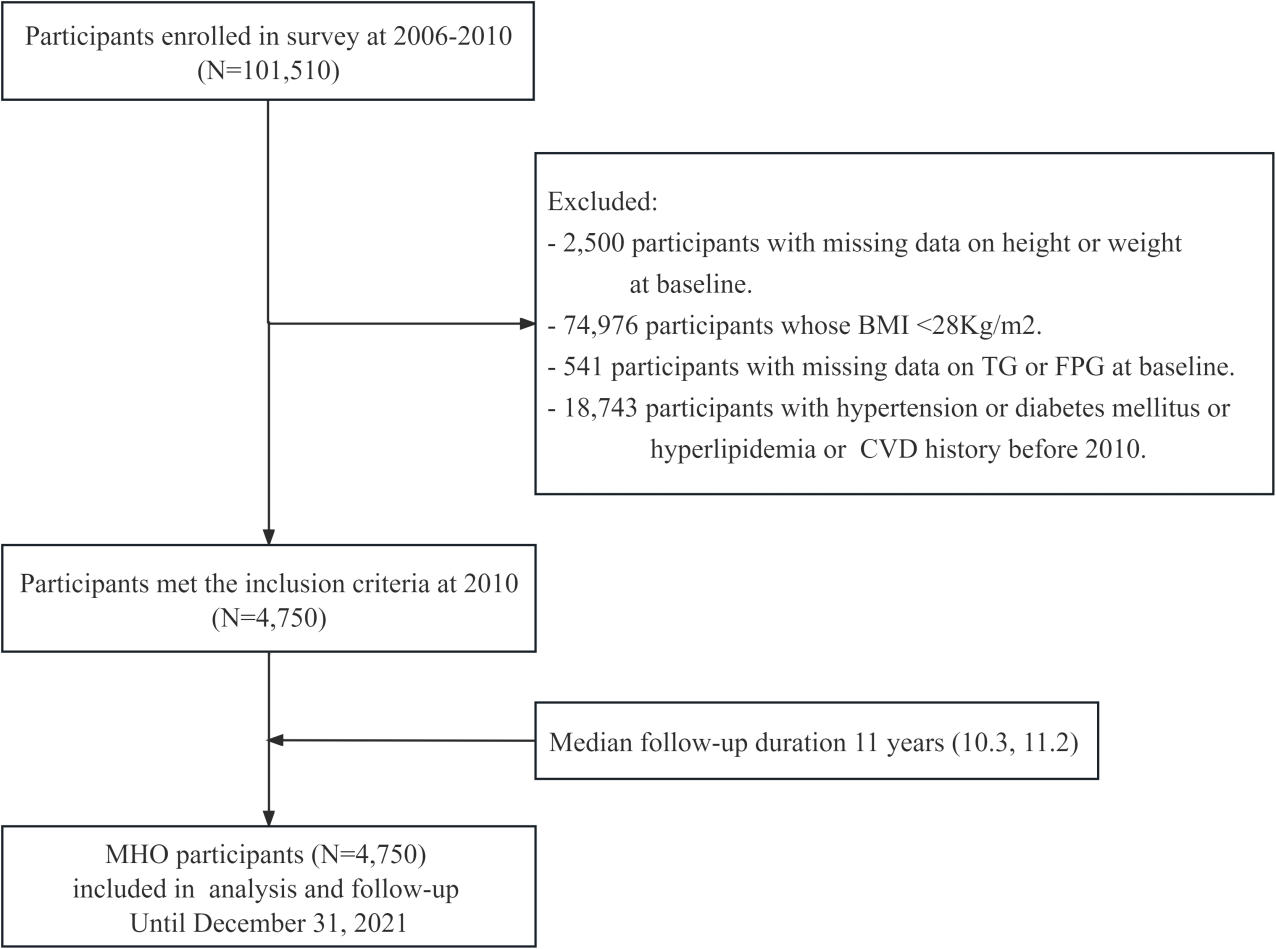
Flow chart for the inclusion of participants in the study.

The Kailuan Study (Registration Number: ChiCTR-TNRC-11001489) is a prospective cohort study based on a community population that investigates cardiovascular diseases and related risk factors through surveys and interventions. The study details can be found in previous publications by our research group (28, 29). In 2006, Kailuan General Hospital and its ten affiliated hospitals conducted initial health examinations for both active and retired employees of the Kailuan Group, with a total of 101,510 participants. Subsequent follow-ups were conducted every two years, maintaining consistency in follow-up content, anthropometric measurements, and biochemical indicators with the initial health examination. This study focused on the metabolically healthy obese (MHO) population, which consisted of obese individuals who participated in the health examinations of the Kailuan Group from 2006 to 2010 and did not have a history of hypertension, diabetes, hyperlipidemia, or cardiovascular disease. The obesity cutoff (BMI ≥28 kg/m²) was defined based on ethnic-specific thresholds accounting for higher visceral adiposity in Asian populations (WHO-recommended BMI ≥27.5 kg/m² for Asians) (30, 31), in alignment with the Chinese Adult Obesity Guidelines (32), while ensuring consistency with previous epidemiological research (33, 34). The specific criteria for inclusion were meeting the obesity criteria (BMI≥28 kg/m^2^) along with the following: serum total cholesterol (TC) <5.72 mmol/L; serum triglycerides (TG) ≤1.7 mmol/L; serum high-density lipoprotein cholesterol (HDL-C) >1.0 mmol/L (males) or >1.3 mmol/L (females); systolic blood pressure (SBP) <140 mmHg and diastolic blood pressure (DBP) <90 mmHg; fasting plasma glucose (FPG) <7 mmol/L; no history of taking antihypertensive, antidiabetic, or lipid-lowering medications; and no history of CVD (including coronary heart disease, ischemic and hemorrhagic stroke, heart failure, atrial fibrillation) (12, 17). Individuals who did not meet these criteria were excluded. A total of 4,750 participants were ultimately included, and follow-up commenced in 2010 and concluded on December 31, 2021. The inclusion and exclusion processes are illustrated in **Figure 1**. The study was performed in accordance with the Declaration of Helsinki and approved by the Ethics Committee of Kailuan General Hospital (Approval Number: 2006-05). All participants provided written informed consent after receiving detailed explanations about the study objectives, data collection procedures, and their right to withdraw at any time without consequences to medical care. This consent process followed the standardized protocol established in the Kailuan cohort, as described in our previous publications (35, 36).

### 2.2 Data collection

This study used the epidemiological survey content and anthropometric indicators mentioned in previously published articles (28, 37). All measurements were conducted in a quiet room with the temperature controlled between 22°C and 25°C. Each participant completed a face-to-face questionnaire during each health examination, collecting information on demographic characteristics (sex, age, education level, BMI), personal health history (hypertension, diabetes, cardiovascular disease, and the use of antihypertensive, glucose-lowering drugs, and lipid-lowering medications), and lifestyle factors (smoking status, alcohol consumption habits, Physical Activity habits), among others. For anthropometric measurements, the participants were instructed to wear lightweight clothing and remain barefoot. Trained doctors measured weight and height under standardized conditions according to a standardized protocol, with units standardized to 0.1 kilograms and 0.1 centimeters. Body mass index (BMI) was calculated as weight (kg) divided by height in meters squared (m^2^). During blood pressure measurements, following a 5-minute rest for participants, calibrated mercury sphygmomanometers were used to measure the blood pressure of each participant’s right upper arm. Measurements were repeated at intervals of 1–2 minutes a total of three blood pressure readings. The average of three readings was recorded (38).

### 2.3 Measurement of biochemical parameters

All participants were required to fast for 8–12 h, and fasting venous blood samples were obtained after centrifugation to collect the upper serum layer for measurement of total cholesterol (TC), low-density lipoprotein cholesterol (LDL-C), high-density lipoprotein cholesterol (HDL-C), triglycerides (TG), fasting plasma glucose (FPG), high-sensitivity C-reactive protein (hs-CRP), and serum uric acid (UA) (37). Biochemical indicators were measured using a Hitachi 7600 automatic biochemical analyzer. The procedures strictly followed the instructions provided with the reagent kits, and the analyses were performed by professional laboratory technicians.

### 2.4 Definitions and grouping of TyG index

The TyG index was calculated using the established formula: ln[TG(mg/dL) × FPG(mg/dL)/2] (37), and it was divided into four groups based on quartiles: Q1 group (<8.18); Q2 group (8.18-8.41); Q3 group (8.42-8.62); Q4 group (≥8.63).

### 2.5 Other defnitions

CVD includes coronary heart disease, ischemic stroke, hemorrhagic stroke, heart failure, and atrial fibrillation (39). Hypertension is defined as systolic blood pressure (SBP) ≥ 140mmHg and/or diastolic blood pressure (DBP) ≥ 90mmHg, or the use of antihypertensive medications, or a history of hypertension (40). Diabetes was defined as fasting plasma glucose (FPG) ≥ 7.0 mmol/L, prolonged use of antidiabetic medications, or a history of a confirmed diabetes diagnosis (41). Hyperlipidemia is defined as total cholesterol (TC) > 5.72 mmol/L or triglycerides (TG) > 1.7 mmol/L, or the prolonged use of statins, niacin, or fibric acid derivatives (42). Participants who smoked an average of ≥1 cigarette per day in the past year were classified as smokers; otherwise, they were considered non-smokers. Participants who had consumed an average of ≥100 mL of alcohol per day (alcohol content >50%) in the past year were classified as drinkers; otherwise, they were considered nondrinkers. Participants engaging in physical exercise ≥3 times per week, with each session lasting ≥30 min, were classified as active exercisers; otherwise, they were classified as non-active exercisers. Educational level was categorized as below high school and high school or above based on the level of education attained (43).

### 2.6 Outcomes

CMD is defined as the occurrence of one or more of the following: diabetes, coronary heart disease, or stroke (3, 4). Using the health examination data from 2010 as the starting point for follow-up and CMD occurrence as the endpoint event, in the case of multiple subtypes of CMD occurring, the subtype of the first event was recorded; for those who did not experience an endpoint event, the follow-up was concluded on December 31, 2021. CMD occurrence was assessed annually by trained medical personnel who reviewed the hospital diagnoses of the study participants in the hospitals affiliated with the Kailuan Group and the designated medical insurance hospitals in Tangshan. All diagnoses were confirmed by professional physicians based on the hospital records.

### 2.7 Statistical analysis

This study utilized SAS 9.4 statistical software (SAS Institute, Inc., Cary, NC, USA) was used for data analysis. In this study, the missingness of baseline covariates was less than 5%. We used Multiple Imputation by Chained Equations (MICE) to handle missing values for subsequent analyses (44). Metric data with a normal distribution were expressed as mean ± standard deviation, and intergroup data were analyzed using one-way analysis of variance. Skewed distribution metric data were represented as medians and quartiles, and intergroup data were analyzed using non-parametric tests (Kruskal-Wallis). Count data were presented as relative numbers or percentages (%), and intergroup comparisons were made using the chi-square test, which was divided into four groups based on quartiles. Prior to conducting Cox analysis, we performed a test for the proportional hazards (PH) assumption (45). The results indicated that the p-value for the PH assumption test was 0.275 (>0.05), suggesting that the proportional hazards assumption was met. The Kaplan–Meier method was used to calculate the incidence rates of CMD and its subtypes (coronary heart disease, stroke, and diabetes) for each group, with comparisons made using the log-rank test. After meeting the proportional hazards assumption, Cox proportional hazards regression models were used. The risk ratio (HR) and 95% confidence interval (95% CI) were calculated to assess the relationship between the TyG index and risk of CMD and its subtypes. Model 1 was unadjusted for other variables, Model 2 adjusted for age (continuous variable, years) and sex (categorical variable, male or female); Model 3, based on Model 2, further adjusted for smoking status (categorical variable, yes or no), drinking habits (categorical variable, yes or no), physical activity habits (categorical variable, active or inactive), education level (categorical variable, senior high school or above), and family history of CMD (categorical variable, present or absent); Model 4, based on Model 3, further adjusted for SBP (continuous variable, mmHg), FPG (continuous variable, mmol/L), HDL-C (continuous variable, mmol/L), LDL-C (continuous variable, mmol/L), hs-CRP (continuous variable, mg/L), BMI (continuous variable, kg/m^2^), UA (continuous variable, μmol/L), and estimated glomerular filtration rate (eGFR, continuous variable, ml/min/1.73m^2^). After adjusting for potential confounding factors, restrictive cubic splines were used to describe the dose-response relationship between TyG index (continuous variables) and the risk of CMD.Subsequently, stratified analyses were conducted based on age (<45 years, ≥45 years), sex (male, female), physical activity habits (active, inactive) and sex-specific median height (above-median, below-median). To ensure the robustness of the results, individuals who developed CMD within 2 years and those with BMI < 30 kg/m^2^ were excluded from the sensitivity analysis of the relationship between the TyG index and risk of CMD. We additionally included waist circumference (≥90 cm for men/≥85 cm for women) as an alternative indicator of central obesity (data from the 2010 follow-up) for sensitivity analysis. Statistical significance was set at *P* < 0.05 (two-tailed) was considered statistically significant.

## 3 Results

### 3.1 Baseline characteristics of participants stratified by quartiles of TyG index

This study included a total of 4,750 MHO participants as the observational population, with an average age of 53.70 ± 12.93 years, and a male proportion of 75.0%. The baseline clinical and biochemical characteristics of the MHO participants, based on the quartiles of the TyG index, are shown in **Table 1**. Stratified by quartiles of the TyG index, compared to the Q1-Q3 groups, the Q4 group had the highest proportion of male participants, highest values for BMI, SBP, DBP, and heart rate indicators, and the poorest metabolic profile (TC, HDL-C, LDL-C, TG, FPG, UA, and hs-CRP). Additionally, the Q4 group exhibited the highest proportion of unhealthy lifestyle habits (smoking, drinking, and lack of exercise) (P < 0.01) (**Table 1**).

**Table 1:** Baseline characteristics of Participants by quartiles of TyG index.

Variables	Overall	Q1	Q2	Q3	Q4	*P* vaule
		<8.18	8.18-8.41	8.42-8.62	≥8.63	
Participants	4750	1187	1188	1186	1189	*/*
Age (years)	53.70 ± 12.93	53.69 ± 13.12	54.08 ± 12.71	52.98 ± 13.12	54.02 ± 12.75	0.14
Male, N (%)	3562 (75.0)	836 (70.4)	895 (75.3)	883 (74.5)	948 (79.7)	<0.01
BMI (kg/m^2^)	28.65 ± 2.50	28.23 ± 2.73	28.55 ± 2.53	28.74 ± 2.35	29.07 ± 2.32	<0.01
SBP (mmHg)	121.71 ± 10.43	120.06 ± 11.09	121.54 ± 10.44	122.47 ± 10.06	122.76 ± 9.91	<0.01
DBP (mmHg)	79.71 ± 6.40	78.46 ± 6.84	79.76 ± 6.34	80.10 ± 6.19	80.50 ± 6.01	<0.01
HR (beats/min)	71.63 ± 9.29	70.87 ± 9.11	71.38 ± 9.18	71.75 ± 9.40	72.51 ± 9.40	<0.01
TyG index	8.38 ± 0.33	7.93 ± 0.19	8.30 ± 0.07	8.52 ± 0.06	8.77 ± 0.10	<0.01
TC (mmmol/L)	4.48 ± 0.70	4.47 ± 0.72	4.64 ± 0.69	4.74 ± 0.68	4.87 ± 0.67	<0.01
HDL-C (mmmol/L)	1.52 ± 0.40	1.59 ± 0.42	1.53 ± 0.39	1.51 ± 0.40	1.45 ± 0.37	<0.01
LDL-C (mmmol/L)	2.60 ± 0.71	2.40 ± 0.71	2.59 ± 0.71	2.65 ± 0.69	2.76 ± 0.69	<0.01
TG (mmmol/L)*	1.10 (0.88-1.35)	0.72 (0.62-0.83)	1.00 (0.91-1.09)	1.21 (1.11-1.32)	1.48 (1.37-1.59)	<0.01
FPG (mmol/L)	5.22 ± 0.61	4.97 ± 0.56	5.12 ± 0.58	5.23 ± 0.56	5.57 ± 0.58	<0.01
hs-CRP (mg/L)*	1.94 (0.75-4.36)	2.01 (0.81-4.40)	1.65 (0.64-3.91)	1.99 (0.70-4.46)	2.17 (0.83-4.55)	<0.01
UA (μmol/L)*	284.59 (229.00-342.33)	280.59 (229.20-335.97)	279.73 (221.37-337.11)	282.00 (227.00-339.00)	294.58 (237.00-354.70)	<0.01
eGFR (ml/min/1.73m^2^)	84.44 ± 19.30	84.67 ± 19.62	83.75 ± 20.26	84.14 ± 18.37	85.18 ± 18.86	0.29
Current smoking, N (%)	1467 (30.9)	327 (27.5)	359 (30.2)	369 (31.1)	412 (34.7)	<0.01
Current drinker, N (%)	1288 (27.1)	298 (25.1)	304 (25.6)	319 (26.9)	367 (30.9)	<0.01
Physical activity, N (%)	628 (13.2)	156 (13.1)	160 (13.5)	161 (13.6)	151 (12.7)	0.92
Education, N (%)	1352 (28.5)	297 (25.0)	334 (28.1)	366 (30.9)	355 (29.9)	<0.01
Family history of CMD, N (%)	103 (2.17)	23 (1.94)	30 (2.53)	28 (2.36)	22 (1.85)	0.62

P-value, comparison of baseline characteristics between quartiles of TyG index.

BMI, body mass index; SBP, systolic blood pressure; DBP, diastolic blood pressure; HR, heart rate; TC, total cholesterol; HDL-C, high-density lipoprotein cholesterol; LDL-C, low-density lipoprotein cholesterol; TG, triglyceride; FPG, fasting plasma glucose; hs-CRP, high-sensitivity C reactive protein; UA, uric acid; eGFR, estimated glomerular filtration rate; Education senior high school or above.

TG*, hs-CRP*, and UA* were skewed distribution variables expressed as medians and quartiles (P25–P75).

### 3.2 Association between TyG index and CMD and its subtypes

**Figure 2:**
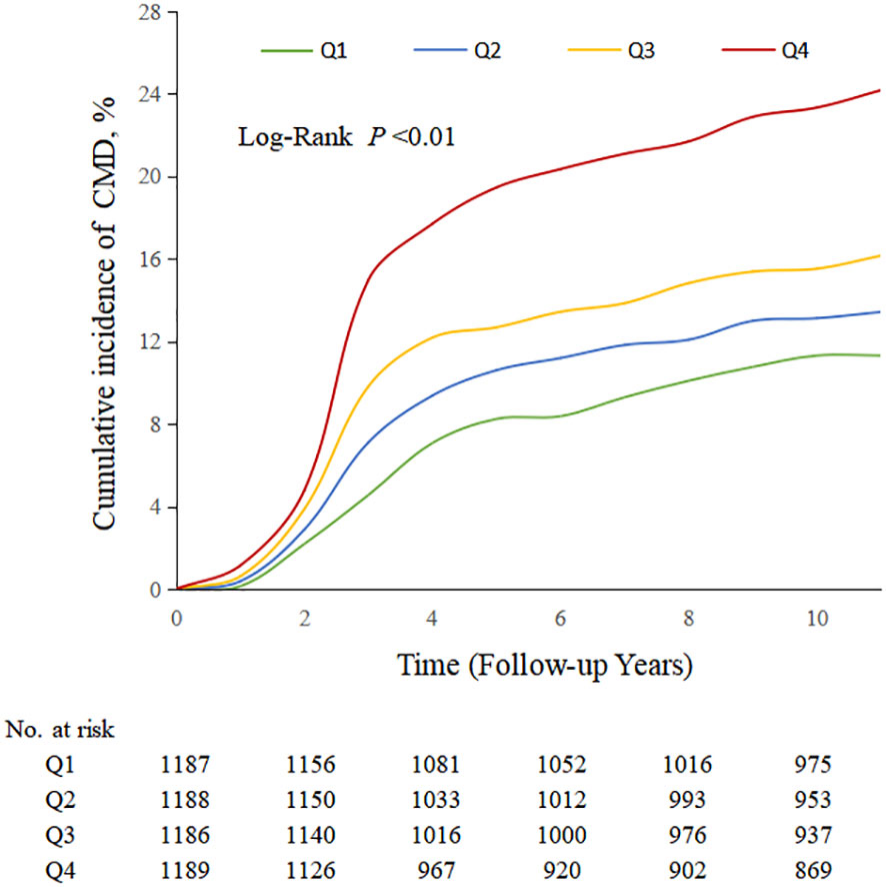
Kaplan–Meier analysis of TyG index and incidence rate of CMD in MHO participants.

**Figure 3:**
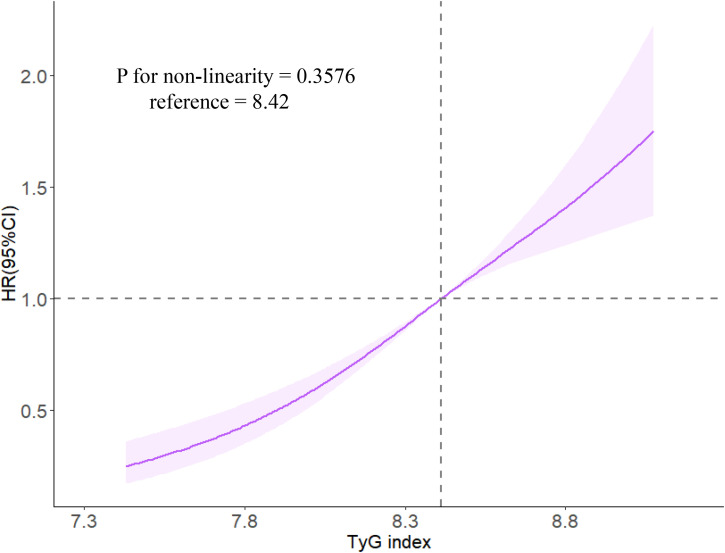
The associations of TyG index with risk of CMD in restrictive cubic spline analysis. Caption: Cox regression models with restricted cubic splines were fitted to the data with three knots at the 10th, 50th, and 90th percentiles of the TyG index. The solid line represents the point estimate of the TyG index with the risk of CMD and the shaded part represents the 95% CI estimate. Covariates in the model included age, sex, smoking status, drinking habits, physical activity habits, education level, family history of CMD, SBP, FPG, HDL-C, LDL-C, hs-CRP, BMI, UA, and eGFR.

During a median follow-up period of 11.4 years (IQR, 10.3, 11.2), 826 (17.39%) MHO participants developed CMD. Among them, 131 MHO participants experienced coronary heart disease, 215 participants had a stroke, and 542 participants developed diabetes. The cumulative incidence rates of CMD subtypes in different TyG quartiles were compared using the log-rank test, which showed statistically significant differences (P < 0.01; **Figure 2**). The incidence density of CMD and its subtypes (coronary heart disease, stroke, and diabetes) exhibited an increasing trend across TyG quartiles. The incidence densities (per 1000 person-years) of CMD in the Q1-Q4 groups were 19.02, 26.13, 27.70, and 45.86, respectively. For coronary heart disease, the densities were 3.36, 3.48, 4.64, and 7.22; for stroke, 4.89, 6.89, 6.99, and 9.78; for diabetes, 11.11, 16.82, 18.43, and 31.27. After adjusting for confounding factors in Model 4, compared to the Q1 group of TyG quartiles, the adjusted HR values (95% CI) for CMD in the Q2-Q4 groups were 1.33 (1.03, 1.65), 1.37 (1.04, 1.82), and 2.04 (1.56, 2.68) (*P* < 0.0001). The adjusted HR values (95% CI) for coronary heart disease were 1.16 (0.88, 1.53), 1.23 (0.92, 1.63), and 1.78 (1.36, 2.33), respectively (*P* < 0.0001). The adjusted HR values (95% CI) for stroke were 1.17 (0.89, 1.55), 1.23 (0.93, 1.64), and 1.75 (1.34, 2.29) (*P* < 0.0001). The adjusted HR values (95% CI) for diabetes were 1.66 (1.18, 2.34), 1.74 (1.23, 2.46), and 2.59 (1.85, 3.63) (*P* < 0.0001). In model 4, for each SD increased, the HR (95% CI) for CMD, coronary heart disease, stroke, and diabetes mellitus was 1.41 (1.27,1.57), 1.35 (1.22,1.50), 1.36 (1.22,1.51) and 1.51 (1.34,1.70), respectively (**Table 2**). Additionally, restrictive cubic spline analysis revealed a linear dose-response relationship between the TyG index and risk of CMD (**Figure 3**).

**Table 2:** Association of TyG index with CMD and subtypes in MHO participants.

Quartiles of TyG index	Case/Total	Incidence density, per 1000 person-years	Model 1	Model 2	Model 3	Model 4
	CMD					
Q1	141/1187	19.02	1.00	1.00	1.00	1.00
Q2	197/1188	26.13	1.43 (1.09,1.88)	1.41 (1.07,1.85)	1.43 (1.08,1.88)	1.33 (1.03,1.65)
Q3	202/1186	27.70	1.46 (1.10,1.92)	1.50 (1.14,1.99)	1.51 (1.15,2.00)	1.37 (1.04,1.82)
Q4	286/1189	45.86	2.29 (1.77,2.97)	2.34 (1.82,3.06)	2.36 (1.82,3.06)	2.04 (1.56,2.68)
*P*-trend			<0.0001	<0.0001	<0.0001	<0.0001
Per 1-SD increase			1.42 (1.28,1.59)	1.44 (1.31,1.62)	1.45 (1.32,1.62)	1.41 (1.27,1.57)
	Coronary Heart Disease					
Q1	26/1187	3.36	1.00	1.00	1.00	1.00
Q2	27/1188	3.48	1.31 (1.00,1.72)	1.31 (0.99,1.72)	1.31 (0.99,1.72)	1.16 (0.88,1.53)
Q3	33/1186	4.64	1.33 (1.01,1.76)	1.43 (1.08,1.89)	1.43 (1.07,1.89)	1.23 (0.92,1.63)
Q4	45/1189	7.22	2.01 (1.55,2.61)	2.16 (1.67,2.81)	2.17 (1.67,2.82)	1.78 (1.36,2.33)
*P*-trend			<0.0001	<0.0001	<0.0001	<0.0001
Per 1-SD increase			1.38 (1.24,1.52)	1.39 (1.25,1.53)	1.39 (1.26,1.53)	1.35 (1.22,1.50)
	Stroke					
Q1	39/1187	4.89	1.00	1.00	1.00	1.00
Q2	51/1188	6.89	1.32 (1.01,1.73)	1.31 (1.00,1.73)	1.32 (1.01,1.73)	1.17 (0.89,1.55)
Q3	54/1186	6.99	1.34 (1.02,1.77)	1.44 (1.09,1.90)	1.44 (1.02,1.90)	1.23 (0.93,1.64)
Q4	71/1189	9.78	2.00 (1.55,2.60)	2.14 (1.65,2.77)	2.13 (1.65,2.78)	1.75 (1.34,2.29)
*P*-trend			<0.0001	<0.0001	<0.0001	<0.0001
Per 1-SD increase			1.37 (1.23,1.52)	1.39 (1.25,1.54)	1.39 (1.25,1.54)	1.36 (1.22,1.51)
	Diabetes mellitus					
Q1	86/1187	11.11	1.00	1.00	1.00	1.00
Q2	130/1188	16.82	1.75 (1.25,2.46)	1.74 (1.24,2.45)	1.76 (1.25,2.47)	1.66 (1.18,2.34)
Q3	131/1186	18.43	1.84 (1.31,2.60)	1.87 (1.33,2.64)	1.88 (1.34,2.65)	1.74 (1.23,2.46)
Q4	195/1189	31.27	2.82 (2.04,3.90)	2.86 (2.06,3.95)	2.87 (2.07,3.97)	2.59 (1.85,3.63)
*P*-trend			<0.0001	<0.0001	<0.0001	<0.0001
Per 1-SD increase			1.53 (1.36,1.72)	1.54 (1.37,1.73)	1.54 (1.37,1.74)	1.51 (1.34,1.70)

Model 1: unjusted.

Model 2: adjusted for age and sex.

Model 3: included variables in model 2 and further smoking status, alcohol consumption habits, physical exercise habits, education, and family history of CMD.

Model 4: included variables in model 3 and further BMI, SBP, FPG, HDL-C, LDL-C, UA, eGFR and hs-CRP.

### 3.3 Results of the stratified analysis and sensitivity analysis

Stratified analysis revealed an interaction between the TyG index and sex, whereas no interactions were found with age, physical activity, and height. Across different age, sex, physical activity, and sex-specific median height subgroups, higher TyG index levels were consistently associated with an increased risk of CMD. Moreover, the risk of CMD in females was higher than that in males. However, in the physically active group, the association between the TyG index and CMD did not show statistically significant differences among the subgroups (**Table 3**).

**Table 3:** Stratified analysis for association of TyG index with CMD in MHO participants.

Subgroup	Event/Total	TyG index				*P* for trend	*P* for interaction
		Q1	Q2	Q3	Q4		
Age							0.5380
<45 years	113/1141	1.00 (Ref.)	1.42 (0.68, 2.98)	2.09 (0.84, 1.74)	2.24 (1.08, 2.56)	0.0103	
≥45 years	727/3609	1.00 (Ref.)	1.18 (0.86, 1.58)	1.21 (0.84, 1.83)	1.66 (1.08, 2.65)	<0.0001	
Sex							0.0029
Female	182/1188	1.00 (Ref.)	1.93 (1.02, 3.63)	2.32 (1.24, 4.34)	4.31 (2.33, 7.97)	<0.0001	
Male	658/3562	1.00 (Ref.)	1.13 (0.82, 1.56)	1.17 (0.86, 1.59)	1.59 (1.17, 2.16)	0.0040	
Physical activity							0.6822
Active	123/628	1.00 (Ref.)	0.89 (0.44, 1.84)	1.10 (0.55, 2.22)	1.82 (0.92, 3.60)	0.1514	
Inactive	717/4122	1.00 (Ref.)	1.44 (0.87, 1.30)	1.45 (0.98, 1.47)	2.17 (1.01, 1.63)	<0.0001	
Height							0.3537
Above-Median	424/2281	1.00 (Ref.)	1.21 (0.81, 1.83)	1.52 (1.03, 2.27)	1.80 (1.20, 2.70)	0.0017	
Below-Median	416/2469	1.00 (Ref.)	1.68 (1.08, 2.64)	1.76 (1.14, 2.70)	2.94 (1.93, 4.47)	<0.0001	

Model adjusted for age, sex, smoking status, alcohol consumption habits, physical exercise habits, education, family history of CMD, BMI, SBP, FPG, HDL-C, LDL-C, UA, eGFR, and hs-CRP.

To eliminate potential confounding effects on the study results, 132 individuals who experienced CMD events within the first 2 years of follow-up (132 individuals) were excluded, and the results were consistent with the main findings (**Supplementary Table S1**). Additionally, sensitivity analysis was performed by repeating the main Cox regression analysis with a focus on individuals with BMI ≥ 30 kg/m^2^, and the results were consistent with the main findings (**Supplementary Table S2**). Waist circumference, as an alternative indicator of MHO to BMI, showed a consistent association pattern with TyG-CMD compared to BMI (**Supplementary Table S4**).

## 4 Discussion

This study found a significant association between TyG index and the risk of CMD in the MHO population. As the TyG index increased, the risk of CMD in MHO individuals also increased. This association remains significant, independent of other risk factors, and the results are consistent across various subtypes of CMD, including coronary heart disease, stroke, and diabetes, consistent with the main findings.

The MHO population is a unique group, and although previous research suggests that MHO is merely a precursor state to metabolically unhealthy obesity (MUO), the risk of CVD remains higher than that in MHNO individuals (16, 46). However, because the MHO population shows no signs of metabolic abnormalities, identifying individuals at a high risk for CMD within this group has become a significant concern. The TyG index serves as an effective indicator reflecting insulin resistance (20, 21). Its effect on the risk of developing hypertension, diabetes, stroke, and CVD in the general population has been confirmed (37, 43, 47, 48). Previous studies from the Kailuan cohort demonstrated that a long-term increase and variation in the TyG index are risk factors for inducing CMD, with a 2.15-fold increase in CMD risk observed in Q4 compared to Q1 of the TyG index (49). Chen et al. found that in a population of young and middle-aged individuals in the United States, obese individuals with a high TyG index had a 1.42-fold increased long-term CVD event risk compared with those with a low TyG index, with no significant difference observed in non-obese individuals (50). The impact of TyG index on CMD and CVD has been studied in obese individuals with metabolic abnormalities (50, 51). Our study discovered that over a median follow-up period of 11 years, 17.39% of MHO participants experienced CMD. Comparing the fourth quartile of the TyG index to the first quartile, the risk of CMD increased by 1.04 times, coronary heart disease risk increased by 78%, stroke risk increased by 75%, and diabetes risk increased by 1.59 times. Our study extends prior evidence by demonstrating that the TyG index retains prognostic value even in the absence of traditional metabolic risk factors. This challenges the notion of MHO as a benign phenotype and underscores the utility of TyG for unmasking latent cardiometabolic risk. Our findings diverge from the Korean NHIS-HealS cohort, which reported no association between the TyG index and cardiovascular events in MHO individuals after full adjustment (51). This discrepancy may arise from methodological differences in the outcome definitions and follow-up duration. Specifically, our composite CMD outcomes included coronary heart disease, stroke, and diabetes, whereas the referenced study by Cho et al. defined cardiovascular events as myocardial infarction and stroke alone. Furthermore, the extended median follow-up period in our cohort (11 years versus 5 years) likely improved the detection of delayed CMD onset, enhancing the statistical power to identify TyG-related risks. Our longitudinal findings challenge the “benign obesity” paradigm, revealing that 17.4% of ostensibly metabolically healthy obese individuals developed cardiometabolic sequelae within a decade. This aligns with the emerging concept of adaptive metabolically healthy obesity proposed by Blüher (15), wherein preserved metabolic parameters mask subclinical endothelial dysfunction. It is crucial to pay close attention to this specific obesity subtype, identify potential risk factors for CMD early, and manage and control them accordingly. However, owing to the lack of apparent metabolic abnormalities in the MHO population, it is challenging to identify individuals at high risk for CMD within this group. The TyG index, as a key indicator of insulin resistance, may help identify individuals at a high risk for CMD in the MHO population by regularly monitoring the TyG index. The dose-response relationship shows each SD of TyG increase confers 41% excess risk, comparable to the impact of 10 mmHg systolic blood pressure elevation on CVD risk (52). As continuous TyG increase associated with 41% higher CMD risk per SD, this supports using TyG as a gradient risk marker rather than binary cut-offs, akin to blood pressure management (53). From a preventive medicine perspective, our findings suggest that lowering TyG index by just 0.5 units (achievable through 15% TG reduction or 0.5 mmol/L FPG decrease) could theoretically reduce CMD risk by 30%.

The clinical implementation of TyG index monitoring in MHO individuals presents notable advantages. As a calculated index derived from routine lipid and glucose measurements, it requires no additional costs beyond standard blood tests—a critical advantage in resource-limited settings. Future studies should validate these estimates in dedicated health economic frameworks.

In the stratified analysis, we found an interaction between the TyG index and sex, with females showing a significantly higher risk of CMD than males. Notably, the sex-specific gradient in TyG-associated risk (4.3-fold higher in females vs. 1.59-fold in males) suggests hormonal or body composition mediators that warrant further investigation. Sex-specific associations may arise from hormonal and genetic factors (54, 55). Estrogen’s insulin-sensitizing effects via AMPK activation could attenuate TyG-related metabolic stress in females (56), though diminished estrogen levels in the predominantly postmenopausal cohort likely reduced this protection, elevating CMD risk. Concurrently, sex differences in the expression levels of metabolism-related genes may be associated with sex differences in gene expression regulation (55). These hypotheses warrant validation using integrated omics approaches. Furthermore, we found that, in the MHO population with inactive physical activity, a higher TyG index was associated with an increased risk of CMD. However, in the MHO population with active physical activity, we observed no statistically significant differences in the risk of CMD among the TyG index groups. Previous research has also demonstrated that maintaining a healthy lifestyle, including higher levels of physical activity, contributes to sustaining MHO in both children and adults (57). A healthy lifestyle is thought to reduce the risk of CMD (4). In addition, sensitivity analyses using the WHO criteria (BMI≥30 kg/m²) showed consistent directionality of the TyG-CMD association.

The mechanism by which TyG index induces CMD in the MHO population is currently unclear. Previous studies have shown that, despite the absence of metabolic disorders in the MHO population, insulin resistance is still significantly higher than that in normal individuals (58). The TyG index, recognized as an effective indicator reflecting insulin resistance (20, 21), is associated with elevated insulin resistance levels as the TyG index increases. Compared with the MHNO population, the MHO group may experience increased visceral fat accumulation, adipose tissue dysfunction, and insulin resistance, potentially placing the body in a process of immune cell infiltration and chronic inflammation. Elevated MASLD prevalence in MHO (25) may further amplify TyG-related risks through hepatokine dysregulation. Specifically, MASLD-driven overproduction of fetuin-A and selenoprotein P impairs adipose tissue insulin signaling (59), creating a feedforward loop between hepatic steatosis and systemic IR. Consequently, pathological conditions, such as hypertension, dyslipidemia, and disrupted blood glucose metabolism, may manifest (60, 61). Furthermore, signals from ectopic fat tissue, including peptide hormones (adipokines) and proinflammatory factors released by immune cells, may accelerate the development of disrupted glucose metabolism, endothelial dysfunction, and atherosclerosis. Under the combined influence of these risk factors, CMD may develop (8, 62–64).

This study is the first prospective cohort study to investigate the TyG index and risk of CMD in the MHO population. However, this study has certain limitations. First, as the study participants were predominantly male employees and retirees of the Kailuan Group, there may be a potential selection bias. Second, although we adjusted for potential confounding factors of CMD, other unmeasured or residual confounding factors such as genetic susceptibility could still influence the results. Third, the MHO participants were from the Kailuan Study, which is a community-based cohort study; therefore, the results of this study cannot be directly extrapolated to other ethnic groups. Fourth, our findings using the Chinese BMI criteria may require validation in populations with different adiposity distributions. Fifth, while TyG index monitoring appears economically viable given its low-cost laboratory basis, we lacked data to perform formal cost-effectiveness analysis. Large-scale implementation would require validation in diverse healthcare systems. Additionally, we made efforts to adjust for confounding factors in the Cox regression model, and the long follow-up period and relatively stable study population enhanced the reliability of our results.

## 5 Conclusions

This study found that in individuals with MHO, the higher the TyG index, the higher the risk of CMD, and this association is independent of other risk factors. Therefore, regular monitoring of the TyG index may help identify MHO individuals at high risk of CMD. Maintaining a lower TyG index may reduce the risk of CMD.

## Data Availability

The datasets used and analyzed during the current study are available from the corresponding author on reasonable request. Requests to access these datasets should be directed to yrchen3@stu.edu.cn.
