# Supplementary material for: Association between triglyceride-glucose index and the risk of cardiometabolic diseases in metabolically healthy obese individuals: a prospective cohort study
Source: Front Endocrinol (Lausanne). 2025 May 19;16:1524786. doi: 10.3389/fendo.2025.1524786 (PMC12127177; doi:10.3389/fendo.2025.1524786)
Supplement: Supplementary file 1 [file Table1.docx]

**Supplemental Material**

Content list:

Table S1 Sensitivity analysis for TyG index with CMD by excluding participants who developed CMD within 2 year during follow-up time (n=132)

Table S2 Sensitivity analysis for TyG index with CMD in participants with BMI≥30Kg/m^2^

Table S3 Comparative Table of MHO Definitions

Table S4 Sensitivity analysis for TyG index with CMD in participants with elevated abdominal circumference as a criterion for MHO

**Table S1** Sensitivity analysis for TyG index with CMD by excluding participants who developed CMD within 2 year during follow-up time (n=132)

| **Quartiles of TyG index** | **Case/Total** | **Incidence density,**  **per 1000 person-years** | **Model 1** | **Model 2** | **Model 3** | **Model 4** |
| --- | --- | --- | --- | --- | --- | --- |
| Q1 | 70/1177 | 9.07 | 1.00 | 1.00 | 1.00 | 1.00 |
| Q2 | 101/1166 | 13.64 | 1.50(1.11,2.03) | 1.48(1.09,2.00) | 1.50(1.11,2.03) | 1.40(1.03,1.90) |
| Q3 | 105/1165 | 14.26 | 1.57(1.16,2.13) | 1.63(1.20,2.21) | 1.64(1.21,2.23) | 1.50(1.10,2.04) |
| Q4 | 132/1110 | 21.36 | 2.31(1.73,3.09) | 2.38(1.78,3.18) | 2.39(1.79,3.30) | 2.11(1.56,2.85) |
| *P*-trend |  |  | <0.0001 | <0.0001 | <0.0001 | <0.0001 |

Model 1: unjusted.

Model 2: adjusted for age and sex.

Model 3: included variables in model 1 and further smoking status, alcohol consumption habits, physical exercise habits, education, and family history of CVD.

Model 4: included variables in model 2 and further BMI, SBP, FPG, HDL-C, LDL-C, UA, eGFR and hs-CRP.

**Table S2** Sensitivity analysis for TyG index with CMD in participants with BMI≥30Kg/m^2^

| **Quartiles of TyG index** | **Case/Total** | **Incidence density,**  **per 1000 person-years** | **Model 1** | **Model 2** | **Model 3** | **Model 4** |
| --- | --- | --- | --- | --- | --- | --- |
| Q1 | 42/247 | 31.62 | 1.00 | 1.00 | 1.00 | 1.00 |
| Q2 | 47/273 | 28.99 | 1.05(0.57,1.94) | 1.02(0.55,1.89) | 1.01(0.54,1.88) | 1.01(0.54,1.88) |
| Q3 | 55/298 | 35.64 | 1.24(0.68,2.27) | 1.26(0.69,2.31) | 1.24(0.68,2.27) | 1.15(0.63,2.11) |
| Q4 | 109/357 | 75.96 | 2.57(1.50,4.39) | 2.54(1.49,4.34) | 2.49(1.46,4.26) | 2.13(1.22,3.73) |
| *P*-trend |  |  | <0.0001 | <0.0001 | <0.0001 | <0.0001 |

Model 1: unjusted.

Model 2: adjusted for age and sex.

Model 3: included variables in model 1 and further smoking status, alcohol consumption habits, physical exercise habits, education, and family history of CVD.

Model 4: included variables in model 2 and further BMI, SBP, FPG, HDL-C, LDL-C, UA, eGFR and hs-CRP.

**Table S3** Comparative table of MHO definitions

| **Study/Criteria** | **BMI Cutoff** | **Metabolic Criteria** | **Exclusion of CVD/Medications** |
| --- | --- | --- | --- |
| Current Study | ≥28 kg/m² | SBP <140mmHg/DBP <90mmHg; FPG <7.0mmol/L; TC<5.72mmol/L; TG ≤1.7mmol/L | Yes |
| Bluher M. [15] | ≥30 kg/m² | ≤1 metabolic syndrome component | No |
| Korean NHIS-HealS [49] | ≥25 kg/m² | No metabolic syndrome | Yes |
| Tsatsoulis A. [44] | ≥30 kg/m² | HOMA-IR <2.5; no hypertension/dyslipidemia | Yes |

**Table S4** Sensitivity analysis for TyG index with CMD in participants with elevated abdominal circumference as a criterion for MHO

| **Quartiles of TyG index** | **Case/Total** | **Incidence density,**  **per 1000 person-years** | **Model 1** | **Model 2** | **Model 3** | **Model 4** |
| --- | --- | --- | --- | --- | --- | --- |
| Q1 | 141/1201 | 18.12 | 1.00 | 1.00 | 1.00 | 1.00 |
| Q2 | 185/1200 | 25.03 | 1.49(1.11,2.02) | 1.40(1.01,1.81) | 1.30(0.91,1.72) | 1.28(0.88,1.68) |
| Q3 | 198/1202 | 26.57 | 1.55(1.14,2.11) | 1.53(1.10,2.01) | 1.48(1.08,1.89) | 1.40(1.05,1.76) |
| Q4 | 257/1202 | 46.66 | 2.21(1.63,2.98) | 2.28(1.68,3.08) | 2.11(1.58,2.64) | 1.96(1.56,2.36) |
| *P*-trend |  |  | <0.0001 | <0.0001 | <0.0001 | <0.0001 |

Model 1: unjusted.

Model 2: adjusted for age and sex.

Model 3: included variables in model 1 and further smoking status, alcohol consumption habits, physical exercise habits, education, and family history of CVD.

Model 4: included variables in model 2 and further BMI, SBP, FPG, HDL-C, LDL-C, UA, eGFR and hs-CRP.
